# [Retracted] MicroRNA‑152 inhibits cell proliferation, migration and invasion by directly targeting MAFB in nasopharyngeal carcinoma

**DOI:** 10.3892/mmr.2023.12960

**Published:** 2023-02-14

**Authors:** Yan Li, Daliu Min, Kai Wang, Shankai Yin, Hongliang Zheng, Liangfa Liu

Mol Med Rep 15: 948–956, 2017; DOI: 10.3892/mmr.2016.6059

Following the publication of this paper, it was drawn to the Editor's attention by a concerned reader that certain of the cell migration and invasion assay data shown in Fig. 3A and B were strikingly similar to data appearing in different form in other articles by different authors at different research institutes, some of which have been retracted; moreover, there appeared to be some overlapping data examining the western blots featured in Figs. 5B and 6A. Owing to the fact that the contentious data in the above article had already been published, or were already under consideration for publication, prior to its submission to *Molecular Medicine Reports*, the Editor has decided that this paper should be retracted from the Journal. After having been in contact with the authors, they agreed with the decision to retract the paper. The Editor apologizes to the readership for any inconvenience caused.

